# A Systematic Review Evaluating Pain Assessment Strategies for Patients With Dementia in the Emergency Department: The Geriatric ED Guidelines 2.0

**DOI:** 10.1111/acem.70230

**Published:** 2026-02-12

**Authors:** Sangil Lee, Alexander X. Lo, Teresita M. Hogan, James D. van Oppen, Cameron J. Gettel, Lauren Lapointe‐Shaw, Justine Seidenfeld, Kaiho Hirata, Márlon Juliano Romero Aliberti, Heather S. Healy, Shan W. Liu, the Geriatric ED Guidelines Writing Group, Scott Dresden, Lauren Cameron Comasco, Angel Li, R. Doreen Monks, Michelle Suh, Luna Ragsdale, Maura Kennedy, Christopher R. Carpenter

**Affiliations:** ^1^ University of Iowa Carver College of Medicine Iowa City Iowa USA; ^2^ Department of Emergency Medicine and Center for Health Services and Outcomes Research Northwestern University Feinberg School of Medicine Chicago Illinois USA; ^3^ Section of Emergency Medicine Section of Geriatrics & Palliative Care University of Chicago Chicago Illinois USA; ^4^ Centre for Urgent and Emergency Care Research University of Sheffield Sheffield UK; ^5^ Department of Emergency Medicine Yale School of Medicine New Haven Connecticut USA; ^6^ Department of Medicine University Health Network and University of Toronto Toronto Canada; ^7^ Center of Innovation to Accelerate Discovery and Practice Transformation, Durham VA Health Care System Durham NC USA; ^8^ International University of Health and Welfare Narita Hospital Narita City Japan; ^9^ Laboratorio de Investigacao Medica Em Envelhecimento (LIM‐66), Servico de Geriatria, Hospital das Clinicas HCFMUSP, Faculdade de Medicina Universidade de São Paulo São Paulo Brazil; ^10^ Research Institute, Hospital Sírio‐Libanês São Paulo Brazil; ^11^ Hardin Library for the Health Sciences University of Iowa Iowa City Iowa USA; ^12^ Department of Emergency Medicine Massachusetts General Hospital, Harvard Medical School Boston Massachusetts USA

**Keywords:** dementia, geriatric emergency department guidelines, pain measurement, systematic review

## Abstract

**Objectives:**

Pain is common among patients presenting to the emergency department (ED) but is frequently underdetected and undertreated in older people living with dementia (PLWD). This systematic review examined whether dementia‐specific pain assessment tools improve pain management compared with usual care in the ED.

**Methods:**

We conducted a systematic review and have reported the methods and results following PRISMA (PROSPERO: CRD420251044828). Eligible studies included randomized, quasi‐experimental, and observational designs enrolling ED patients aged ≥ 65 years with dementia or cognitive impairment. Interventions were pain assessment tools developed for PLWD, and comparisons were with standard pain scales. Primary outcomes were patient‐reported outcome measures and analgesia administration; secondary outcomes included repeated pain scores, ED revisits, functional decline, mortality, and adverse events. Five databases (Ovid MEDLINE, Embase, Cochrane Library, CINAHL, PsycInfo) and two clinical trial registries were searched without language or date restrictions on April 22, 2025, and December 16, 2025, respectively. Two reviewers independently screened, extracted data, and assessed risk of bias using Cochrane RoB‐2.

**Results:**

Of 987 records identified, 18 underwent full‐text review, and one study met eligibility criteria. Fry et al. (2017) conducted a multicenter, cluster‐randomized controlled trial of 602 older adults with suspected long bone fractures, comparing the Pain Assessment in Advanced Dementia (PAINAD) tool with standard pain scales. No significant differences were observed in median time to first analgesia (83 vs. 82 min, *p* = 0.42) or proportion receiving analgesia within 60 min (28% vs. 32%, *p* = 0.19). Evidence certainty was rated very low.

**Conclusions:**

Evidence on dementia‐specific pain assessment tools in the ED is extremely limited. Available data suggest PAINAD does not improve timeliness of analgesia, underscoring the urgent need for rigorous studies to guide pain management for PLWD in the ED.

## 1 Introduction

Approximately 4 million older patients present to United States emergency departments with acute pain severe enough to require analgesic administration. Older adults report moderate to severe pain in 40%–50% of all emergency department (ED) visits, and about 50% of people living with dementia (PLWD) have chronic pain [1]. However, due to communication, reporting, and assessment issues, pain is often underdetected and undertreated among people with dementia [2]. Appropriate pain management is essential for proper medical care, is expected by patients and family members, is a quality metric, and an ethical/moral imperative. In addition, pain is associated with delirium as well as agitation, which is a part of behavioral and psychological symptoms of dementia (BPSD) [3]. Therefore, detecting and treating pain in people with dementia is of great importance for their safety and quality of life.

Subjective pain assessment tools such as the Numerical Rating Scale, which is a gold standard for patients without dementia, may not be appropriate for people with more advanced dementia because of their impaired cognitive and verbal communication skills [4]. Furthermore, objective or observational pain assessment can be challenging as BPSD overlaps with many common behavioral signs of pain, such as restlessness, agitation, and aggression [3]. Therefore, a holistic approach is necessary for assessing pain in people with dementia, and a variety of pain assessment tools, including PAINAD, until now [5, 6].

Therefore, a holistic approach is necessary for assessing pain in people living with dementia, and a variety of pain assessment tools, including PAINAD, were reported to date [5, 6]. The development and validation of these scales for PLWD in the ED setting remain limited. There is no consensus to assess pain in PLWD due to a lack of comprehensive evidence [7, 8]. Also, most EDs lack proper pain management, such as pain assessment, reassessment, time to treatment, type of treatment, and result of treatment. We aimed to systematically review and synthesize the literature on pain assessment tools for PLWD seen in the emergency department. By comparing those pain assessment tools, we aim to achieve improved pain management for patients with dementia in the ED.

## 2 Methods

### 2.1 Study Overview

This was a systematic review and meta‐analysis of pain assessment tools for people living with dementia (PLWD). The study protocol was registered on PROSPERO (CRD 420251044828).

### 2.2 Eligibility Criteria

The eligibility criteria cover aspects related to the population, intervention, control, study design, and outcomes.

#### 2.2.1 Study Population

The population of interest was people aged 65 or older, living with dementia, presenting to the ED. Dementia included mild cognitive impairment, Alzheimer's disease and related dementias (ADRD). More than 70% of study participants needed to have dementia or ADRD to be included. More than 70% of study participants had dementia or ADRD. Dementia was identified based on the past history, collateral history, risk stratification or assessment tools. Studies involving non‐human, simulation, non‐ED settings, or outpatient, hospital, and ICU locations as a source of the index encounter were excluded.

#### 2.2.2 Intervention

The use of one or more pain assessment tools designed for PLWD in the ED.

#### 2.2.3 Control

The control population was defined as those who received usual care, including the standard pain scale or visual analogue scale. There were no exclusion criteria, and studies that did not report comparison groups were included in this review.

#### 2.2.4 The Type of Study

We included randomized controlled trials, quasi‐experimental, cohort, cross‐sectional, pre‐ and post‐study designs, as well as quality improvement projects. We excluded case reports, case series (*n* up to 5), scoping/systematic and narrative reviews, and qualitative studies.

#### 2.2.5 Outcomes

The primary outcome was patient‐reported outcome measures (PROMs), which collect information directly from patients about health status, symptoms, quality of life, and functions. Secondary outcomes were medications given for analgesia (NSAIDs, opioids, GABA agonists), improvement on pain scale after intervention (repeated pain scores), ED revisits, falls, functional decline, mortality, waiting time (ED LOS, days in hospital, admission rate), sedation in the ED, and agitation in the ED.

### 2.3 Information Sources and Search Strategy

Search strategies using subject headings and keywords were designed by a health sciences librarian with input on concepts and terms from the team. Elements from related searches were incorporated [9]. An initial search strategy was developed for Ovid MEDLINE. The keyword portion of the strategy was translated for the other databases with the aid of the Polyglot search translator and checked for accuracy [9]. Subject headings for each database were manually selected. No date or language limits were applied. Five databases (Ovid MEDLINE, Embase, Cochrane Library, CINAHL, PsycInfo) and two clinical trial registries were searched without language or date restrictions on April 22, 2025 and December 16, 2025 respectively. Database results were combined in EndNote and duplicates were removed through automated and manual methods. Search strategies for all databases are available in the supplemental materials (Appendix S1).

### 2.4 Selection Process

The review team health science librarian uploaded the database search results to Covidence. Any duplicate entries were removed in EndNote and in Covidence. Two reviewers independently screened for titles and abstracts. We resolved disagreements through review by a third independent reviewer or through discussion in the review team meeting. The same methods for screening and adjudicating were used for full manuscript review.

### 2.5 Data Collection

We used a standardized data collection form to extract data. Two independent reviewers extracted data from each study. If there was a discrepancy, a third person reviewed the study or we discussed the discrepancy in the review team meeting. We extracted the following study characteristics: author, year, design and number of sites, country, study sample characteristics, dementia assessment tool (index or reference test), age (average, SD or IQR), sample size, % male/female, dementia prevalence (%), stage of dementia (if available, descriptive), type of dementia, place of living, comorbidities, frailty scale, and outcome type(s) and result(s) with measures of uncertainty around estimates.

### 2.6 Risk of Bias Assessment

The Cochrane risk of bias tool (version 2) for RCT was used for this systematic review. We planned to use the New‐Castle Ottawa scale for non‐RCT study. Two independent raters assessed the quality of each study. Disagreements were resolved through discussion or through the review team meeting until a consensus was achieved.

### 2.7 Effect Measures

We planned to summarize and report absolute/relative risk, odds ratio, risk difference, hazard ratio, and/or number needed to treat for binary outcomes, along with measures of uncertainty (e.g., 95% confidence intervals) around estimate, as available from the original publications. Any score, scale, and time of administration were reported for continuous outcome variables.

### 2.8 Synthesis

If more than 2 high‐quality studies with homogeneous intervention/outcome data were available, we planned to perform a meta‐analysis for either continuous variable or categorical variables as outcomes. However, the per‐protocol meta‐analysis was not undertaken due to limited evidence. Instead, a narrative summary was provided.

### 2.9 Certainty Assessment

The GRADE certainty assessment will be completed in a separate Dementia Guideline manuscript similar to other Geriatric Emergency Department Guidelines 2.0 [10].

## 3 Results

### 3.1 Study Selection

**FIGURE 1:**
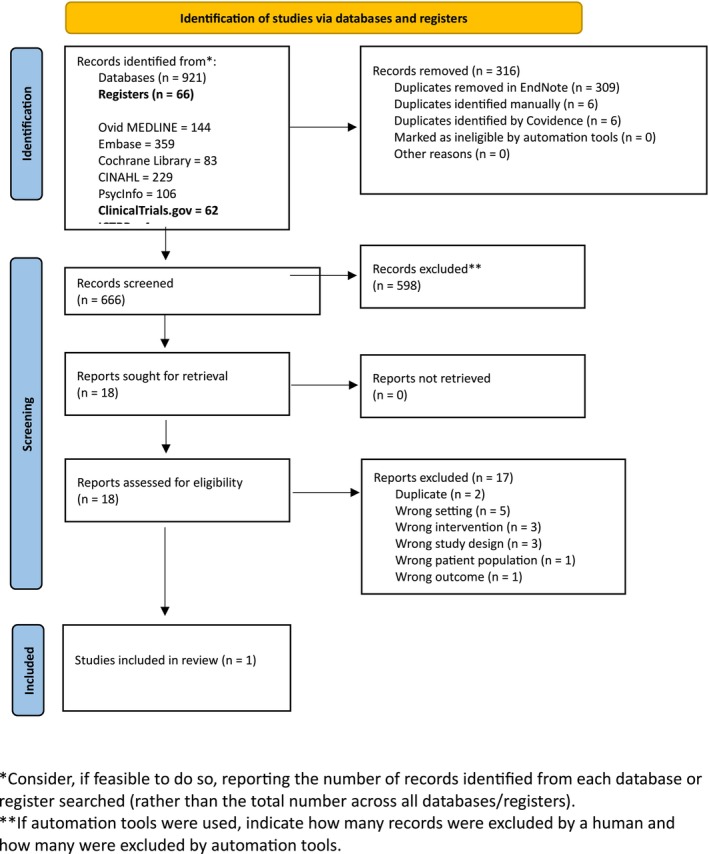
PRISMA flow diagram. *Consider, if feasible to do so, reporting the number of records identified from each database or register searched (rather than the total number across all databases/registers). **If automation tools were used, indicate how many records were excluded by a human and how many were excluded by automation tools.

A total of 921 records were identified through database searches. After removing duplicates, 605 records underwent title and abstract screening. Of these, 587 were excluded for ineligibility. The remaining eighteen full‐text articles were assessed for eligibility, of which one study met the inclusion criteria and was included in the final review (Figure 1).

### 3.2 Study Characteristics

The demographic characteristics of the included study are summarized in Table 1. The study, conducted by Fry et al. (2017) in Australia, was a multicenter, cluster‐randomized, controlled trial involving eight emergency departments. Dementia or cognitive impairment was identified using the Six‐Item Screener (SIS) among all of the older ED adults recruited. The intervention of interest was the Pain Assessment in Advanced Dementia (PAINAD) tool, evaluated for older adults (≥ 65 years) with suspected long bone fractures. Details on intervention and outcome measures are presented (Table 1). As the review identified only one eligible study, a meta‐analysis could not be performed.

**TABLE 1:** Study characteristics and key outcomes.

Author, year	Country/setting	Design/sites	Population	Dementia tool	Intervention	Comparator	Primary outcomes	Admission rate
Fry (2017)	Australia, 8EDs	Multicenter cluster RCT	Older than 65 with cognitive impairment and suspected long bone fracture *M* = 602; median age 83–86; 71%–74% female; 45% with dementia	Six‐Item Screener (SIS less than 4)	PAINAD tool	Standard pain scales	Median time to analgesia: 83 vs. 82 min (*p* = 0.42); Analgesia less than 60 min: 28% vs. 32% (*p* = 0.19)	86% both groups; CI subgroup: 87% vs. 91% (*p* = 0.255)

### 3.3 Results of Included Study

The included study did not provide patient‐reported outcome measures, which were the primary outcome of interest in this review.

Fry et al. [11] was a multicenter cluster randomized controlled trial that enrolled 602 older adults presenting with suspected long bone fractures, in whom cognitive impairment was identified with the SIS. Patients at intervention sites were assessed using the PAINAD tool, while those at control sites used standard pain scales. The authors did not find significant differences in the median time from ED arrival to administration of the first analgesic dose between groups (83 min in the PAINAD group vs. 82 min in the control group, *p* = 0.42). Similarly, the proportion of patients receiving analgesia within 60 min did not differ (28% vs. 32%, *p* = 0.19). A subgroup analysis restricted to patients with SIS‐defined impairment suggested a nonsignificant reduction of 13 min in time to first analgesia in the intervention group (90 vs. 103 min, *p* = 0.62). While the use of PAINAD did not result in measurable improvements in the timeliness of pain treatment, the study demonstrated persistent challenges in optimizing analgesia delivery for cognitively impaired older adults in the ED.

### 3.4 Risk of Bias in Studies

**FIGURE 2:**
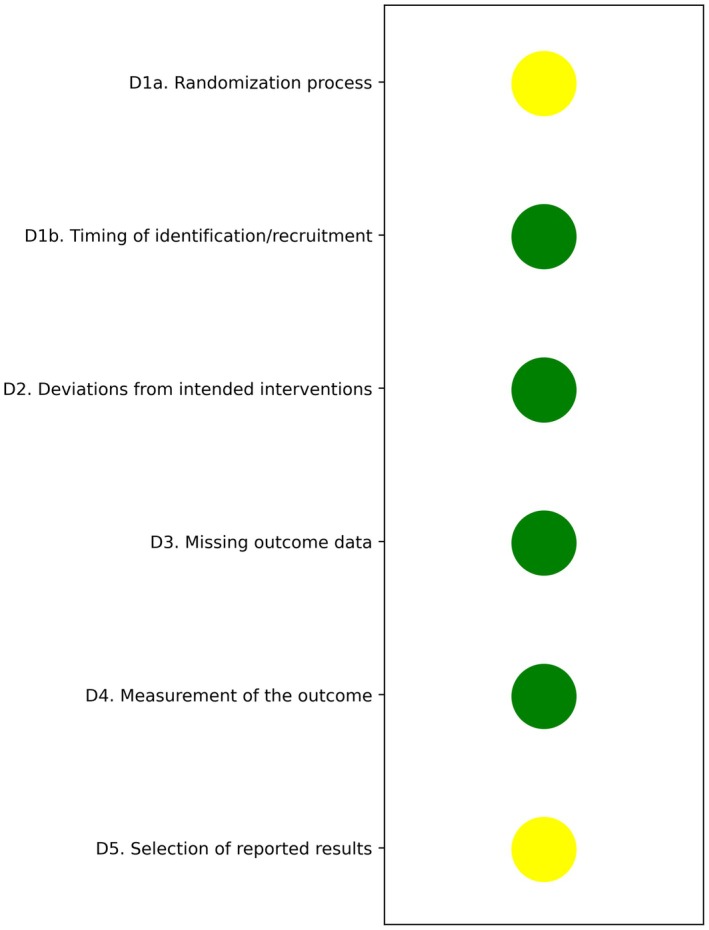
Risk of bias.

The cluster‐randomized controlled trial was evaluated using the Cochrane RoB‐2 tool and was judged to have some concerns, particularly regarding the comparability of groups and outcome assessment, although participant selection was deemed appropriate (Figure 2). Because the evidence relied on a single study with methodological shortcomings and was restricted to a narrow geographic setting, the overall certainty of the evidence would likely be rated very low according to GRADE, pending a voting process.

## 4 Discussion

In this systematic review, we identified one study using the PAINAD scale for PLWD but there was no significant improvement in measures of clinical care. The overall lack of studies indicates a significant existing gap in the literature and potentially our clinical care for PLWD who experience pain in the emergency department.

Fry's randomized clinical trial on the use of the PAINAD pain scale and its impact on the timing of analgesia administration failed to show a significant reduction in the timing of analgesia administration [11]. Nonetheless, the study offers an important blueprint for the feasibility and conduct of future studies looking at pain measurement and pain management among PLWD in the ED. Fry and colleagues restricted their study to patients with long‐bone fractures. As such, participants may have received timely analgesia regardless of their cognitive impairment history as these injuries are widely recognized as being painful and requiring urgent analgesia. While this patient selection was intentional to ensure a group with what the investigators described as significant pain and thus minimize the potential misclassification of pain, it substantially limits the generalizability of the study results to PLWD with pain from other causes such as headache, chest pain, abdominal pain, or extremity pain from nontraumatic causes. The PAINAD scale [6] is a well‐documented pain measurement scale among PLWD, and its psychometric properties, validity and reliability involving English and non‐English language applications have been extensively examined in multiple patient populations, except specifically the ED patient populations [12, 13]. Our systematic review limits any extrapolation of the results to other pain scales aside from the PAINAD that were developed for PLWD [12, 13].

The paucity of studies within the specific context of the identification and measurement of pain among PLWD in the ED setting underscores a significant evidence gap for a growing patient population [14] and their anticipated increased utilization of the ED [15]. This systematic review reiterates others' conclusions on the limited evidence to guide care of PLWD in ED settings. In 2022, the National Institute on Aging‐funded Geriatric Emergency care Applied Research (GEAR) Network 2.0—Alzheimer's Dementia Care Network interdisciplinary working group and consensus conferences identified several key evidence gaps and areas in need of urgent care improvement for PLWD, including research on optimal ED care, care transitions after ED visits, patient communication, and screening for dementia in the ED setting [16, 17, 18].

The clinical implication of the findings is that the assessment of pain among PLWD in the ED is poor and without an evidence base to guide practice improvement. Consequently, one could reasonably predict that repeat ED visits among PLWD for pain will persist, but the inability to properly assess pain may contribute to poor management of pain in PLWD, and poor outcomes such as prolonged ED stays and/or return ED visits may be more likely to occur. Pain is one of the leading drivers of ED utilization by PLWD, and even more so at the end of life [19], and while the PAINAD scale offers a reasonable tool for the ED [6], there is a lack of robust studies to guide its use and implementation in the ED. As is typical of ED encounters, the unfamiliarity with the patient and the fragmented information on the etiology and history of the pain contribute to the challenge of pain management [20]. The successful management of acute pain among PLWD has been demonstrated in other settings, but the implementation of any pain intervention in the ED will depend on the valid and reliable assessment of pain, which is currently lacking. Future studies should include other methods of pain ascertainment and measurement for PLWD in the ED, including the assessment of the impact of tools using caregivers to report pain in PLWD prior to their arrival in the ED [21] and among non‐English speaking PLWD in the ED [22]. The next steps could be to apply another scale from other conditions of populations where communication and cognition are challenges, for example, the use of visual aid to estimate the pain severity.

## 5 Limitations

This systematic review has some important considerations. First, despite using the standard method to screen for inclusion and exclusion, it is possible that our systematic review did not identify all relevant studies. Second, although we conducted a comprehensive search across multiple databases, only one study met our inclusion criteria. While this limits the breadth of evidence available, it also underscores the novelty of this research area and the opportunity for future investigation. Third, the included study focused specifically on the PAINAD tool in patients with long bone fractures in Australia. This context may not fully reflect other clinical populations or settings, suggesting that additional research is needed to establish generalizability. Finally, as with most reviews, publication bias remains a possibility since studies with null or negative findings are less likely to appear in the published literature.

## 6 Conclusion

This systematic review highlights a significant evidence gap regarding dementia‐specific pain assessment tools in the emergency department. Only one multicenter cluster‐randomized controlled trial met eligibility criteria, and it evaluated the use of PAINAD in patients with long bone fractures. While this trial did not demonstrate differences in analgesia administration compared with standard pain scales, its findings provide a valuable starting point for understanding the challenges of pain assessment in this population. Given the high prevalence of pain among persons living with dementia and the risks associated with undertreatment, there is an urgent need for high‐quality research to develop, adapt, and validate dementia‐specific pain assessment tools in ED settings. Such efforts are essential to advancing equitable, patient‐centered emergency care for this vulnerable and growing population.

## Supporting information


**Appendix S1:** Search strategies.

## Data Availability

The data that support the findings of this study are available on request from the corresponding author. The data are not publicly available due to privacy or ethical restrictions.

## Appendix A Geriatric ED Guidelines Dementia Writing Group

Scott Dresden, MD, Associate Professor, Department of Emergency Medicine, Northwestern University Feinberg School of Medicine, Chicago, IL; Lauren Cameron Comasco, MD, Assistant Professor, Department of Emergency Medicine, Corewell Health William Beaumont University Hospital, Oakland University William Beaumont School of Medicine, Royal Oak, MI, lauren.cameron-comasco@corewellhealth.org; Angel Li, MD, MBA, Assistant Professor, Department of Emergency Medicine, The Ohio State University, angel.li@osumc.edu; R. Doreen Monks, RN (retired), MSN, Board Member, Voice of Alzheimer's, Washington DC, monksdr@gmail.com; Michelle Suh, MD, Brown University School of Medicine, Providence, RI, misuhmed@gmail.com; Luna Ragsdale, MD, MPH, Chief, Emergency Department, Durham VA Healthcare System, Clinical Associate, Department of Emergency Medicine, Duke University Hospital, luna.ragsdale@va.gov; Justine Seidenfeld, MD, MHS, Core Investigator, Center of Innovation to Accelerate Discovery and Practice Transformation, Durham VA Health Care System, justine.seidenfeld@va.gov; Maura Kennedy, MD, MPH, Associate Professor, Department of Emergency Medicine, Massachusetts General Hospital, Harvard Medical School, mkennedy8@mgb.org; Christopher R. Carpenter, MD, MSc, Professor, Department of Emergency Medicine, Mayo Clinic, Rochester, MN, carpenter.christopher@mayo.edu, ORCID ID: 0000‐0002‐2603‐7157.
