# Supplementary material for: A Systematic Review Evaluating Pain Assessment Strategies for Patients With Dementia in the Emergency Department: The Geriatric ED Guidelines 2.0
Source: Acad Emerg Med. 2026 Feb 12;33(2):e70230. doi: 10.1111/acem.70230 (PMC12896694; doi:10.1111/acem.70230)
Supplement: Supplementary file 1 — Appendix S1: Search strategies. [file ACEM-33-0-s001.docx]

**Appendix 1. Search Strategies**

**Ovid MEDLINE**

(exp Dementia/ or ((alzheimer* or diffuse cortical sclerosis or amentia* or cadasil or cadasilm or (cerebral arteriopathy adj2 subcortical infarcts) or cerebral autosomal dominant arteriopathy or ((basal cortical or basalcortical or baso cortical or basocortical or cortical basal or corticalbasal or cortico basal or corticobasal) adj2 (degeneration or syndrome)) or CBD or CBGD or creutzfeldt jakob or CJD or ((cognit* or neurocognit* or frontotemporal) adj2 (disorder* or defect* or deficit* or decline* or deteriorat* or disabilit* or dysfunction* or disfunction* or impair* or interference*)) or spongiform encephalopath* or dementia* or demention or ((aids or hiv) adj3 (encephalopath* or motor complex)) or huntington* or kluver bucy syndrome or temporal lobectomy behavior syndrome or lewy bod* or lewybod* or pick* complex or pick* disease or pick* syndrome or presenile or prion* disease* or pseudodementia or rett* disease or rett* syndrome or morbus rett or senile or senilitas or senility or tauopathy*).ti,ab,kf.)) AND (Emergency Treatment/ or Emergency Medical Services/ or exp Emergency Room Visits/ or exp Emergency Service, Hospital/ or Emergency Services, Psychiatric/ or Emergency Medicine/ or exp Emergency Nursing/ or exp Evidence-Based Emergency Medicine/ or ((acute care or acute medical care or (("a and e" or "a-e" or ae or casualty or emergency or trauma) adj1 (center* or centre* or department* or room* or service* or system* or unit* or ward*)) or ED or ER or ((emergency or trauma) adj2 (care or doctor* or medicine or nurs* or patient* or physician* or personnel or provider* or psychiatr* or specialist or treatment*)) or emergentologist or emergicenter or psychiatric EMS).ti,ab,kf.)) AND (exp Pain Measurement/ or exp Pain Management/ or ((grimace scale or ((pain or analgesia) adj1 (assess* or intensity* or inventor* or measure* or question* or scale* or scor* or severity or test*)) or nociception test* or pain acceptance questionnaire or painDETECT or (VAS adj3 pain) or (visual analog* scale and pain) or pain management).ti,ab,kf.))

**Embase**

('dementia'/exp OR (alzheimer*:ti,ab,kw OR 'diffuse cortical sclerosis':ti,ab,kw OR amentia*:ti,ab,kw OR cadasil:ti,ab,kw OR cadasilm:ti,ab,kw OR (('cerebral arteriopathy' NEAR/2 'subcortical infarcts'):ti,ab,kw) OR 'cerebral autosomal dominant arteriopathy':ti,ab,kw OR ((('basal cortical' OR basalcortical OR 'baso cortical' OR basocortical OR 'cortical basal' OR corticalbasal OR 'cortico basal' OR corticobasal) NEAR/2 (degeneration OR syndrome)):ti,ab,kw) OR cbd:ti,ab,kw OR cbgd:ti,ab,kw OR 'creutzfeldt jakob':ti,ab,kw OR cjd:ti,ab,kw OR (((cognit* OR neurocognit* OR frontotemporal) NEAR/2 (disorder* OR defect* OR deficit* OR decline* OR deteriorat* OR disabilit* OR dysfunction* OR disfunction* OR impair* OR interference*)):ti,ab,kw) OR 'spongiform encephalopath*':ti,ab,kw OR dementia*:ti,ab,kw OR demention:ti,ab,kw OR (((aids OR hiv) NEAR/3 (encephalopath* OR 'motor complex')):ti,ab,kw) OR huntington*:ti,ab,kw OR 'kluver bucy syndrome':ti,ab,kw OR 'temporal lobectomy behavior syndrome':ti,ab,kw OR 'lewy bod*':ti,ab,kw OR lewybod*:ti,ab,kw OR 'pick* complex':ti,ab,kw OR 'pick* disease':ti,ab,kw OR 'pick* syndrome':ti,ab,kw OR presenile:ti,ab,kw OR 'prion* disease*':ti,ab,kw OR pseudodementia:ti,ab,kw OR 'rett* disease':ti,ab,kw OR 'rett* syndrome':ti,ab,kw OR 'morbus rett':ti,ab,kw OR senile:ti,ab,kw OR senilitas:ti,ab,kw OR senility:ti,ab,kw OR tauopathy*:ti,ab,kw)) AND ('emergency treatment'/de OR 'emergency care'/de OR 'evidence based emergency medicine'/exp OR 'emergency health service'/de OR 'emergency ward'/exp OR 'hospital emergency service'/exp OR 'psychiatric emergency service'/de OR 'emergency physician'/exp OR 'emergency department visit'/exp OR 'emergency nursing'/exp OR 'emergency nurse practitioner'/exp OR 'emergency nurse'/exp OR ('acute care':ti,ab,kw OR 'acute medical care':ti,ab,kw OR ((('a and e' OR 'a e' OR ae OR casualty OR emergency OR trauma) NEAR/1 (center* OR centre* OR department* OR room* OR service* OR system* OR unit* OR ward*)):ti,ab,kw) OR ed:ti,ab,kw OR er:ti,ab,kw OR (((emergency OR trauma) NEAR/2 (care OR doctor* OR medicine OR nurs* OR patient* OR physician* OR personnel OR provider* OR psychiatr* OR specialist OR treatment*)):ti,ab,kw) OR emergentologist:ti,ab,kw OR emergicenter:ti,ab,kw OR 'psychiatric ems':ti,ab,kw)) AND ('pain assessment'/exp OR 'pain assessment in advanced dementia'/exp OR 'pain assessment in advanced dementia scale'/exp OR 'analgesia'/exp OR ('grimace scale':ti,ab,kw OR 'pain management':ti,ab,kw OR (((pain OR analgesia) NEAR/1 (assess* OR intensity* OR inventor* OR measure* OR question* OR scale* OR scor* OR severity OR test*)):ti,ab,kw) OR 'nociception test*':ti,ab,kw OR 'pain acceptance questionnaire':ti,ab,kw OR paindetect:ti,ab,kw OR ((vas NEAR/3 pain):ti,ab,kw) OR ('visual analog* scale':ti,ab,kw AND pain:ti,ab,kw)))

**Cochrane CENTRAL**

([mh Dementia] OR (alzheimer*:ti,ab,kw OR "diffuse cortical sclerosis":ti,ab,kw OR amentia*:ti,ab,kw OR cadasil:ti,ab,kw OR cadasilm:ti,ab,kw OR ("cerebral arteriopathy":ti,ab,kw NEAR/2 "subcortical infarcts":ti,ab,kw) OR "cerebral autosomal dominant arteriopathy":ti,ab,kw OR (("basal cortical":ti,ab,kw OR basalcortical:ti,ab,kw OR "baso cortical":ti,ab,kw OR basocortical:ti,ab,kw OR "cortical basal":ti,ab,kw OR corticalbasal:ti,ab,kw OR "cortico basal":ti,ab,kw OR corticobasal:ti,ab,kw) NEAR/2 (degeneration:ti,ab,kw OR syndrome:ti,ab,kw)) OR CBD:ti,ab,kw OR CBGD:ti,ab,kw OR "creutzfeldt jakob":ti,ab,kw OR CJD:ti,ab,kw OR ((cognit*:ti,ab,kw OR neurocognit*:ti,ab,kw OR frontotemporal:ti,ab,kw) NEAR/2 (disorder*:ti,ab,kw OR defect*:ti,ab,kw OR deficit*:ti,ab,kw OR decline*:ti,ab,kw OR deteriorat*:ti,ab,kw OR disabilit*:ti,ab,kw OR dysfunction*:ti,ab,kw OR disfunction*:ti,ab,kw OR impair*:ti,ab,kw OR interference*:ti,ab,kw)) OR ("spongiform" NEXT encephalopath*):ti,ab,kw OR dementia*:ti,ab,kw OR demention:ti,ab,kw OR ((aids:ti,ab,kw OR hiv:ti,ab,kw) NEAR/3 (encephalopath*:ti,ab,kw OR "motor complex":ti,ab,kw)) OR huntington*:ti,ab,kw OR "kluver bucy syndrome":ti,ab,kw OR "temporal lobectomy behavior syndrome":ti,ab,kw OR ("lewy" NEXT bod*):ti,ab,kw OR lewybod*:ti,ab,kw OR (pick* NEXT "complex"):ti,ab,kw OR (pick* NEXT "disease"):ti,ab,kw OR (pick* NEXT "syndrome"):ti,ab,kw OR presenile:ti,ab,kw OR (prion* NEXT disease*):ti,ab,kw OR pseudodementia:ti,ab,kw OR (rett* NEXT "disease"):ti,ab,kw OR (rett* NEXT "syndrome"):ti,ab,kw OR "morbus rett":ti,ab,kw OR senile:ti,ab,kw OR senilitas:ti,ab,kw OR senility:ti,ab,kw OR tauopathy*:ti,ab,kw)) AND ([mh ^"Emergency Treatment"] OR [mh ^"Emergency Medical Services"] OR [mh "Emergency Room Visits"] OR [mh "Emergency Service, Hospital"] OR [mh ^"Emergency Services, Psychiatric"] OR [mh ^"Emergency Medicine"] OR [mh "Emergency Nursing"] OR [mh "Evidence-Based Emergency Medicine"] OR ("acute care":ti,ab,kw OR "acute medical care":ti,ab,kw OR (("a and e":ti,ab,kw OR a-e:ti,ab,kw OR ae:ti,ab,kw OR casualty:ti,ab,kw OR emergency:ti,ab,kw OR trauma:ti,ab,kw) NEAR/1 (center*:ti,ab,kw OR centre*:ti,ab,kw OR department*:ti,ab,kw OR room*:ti,ab,kw OR service*:ti,ab,kw OR system*:ti,ab,kw OR unit*:ti,ab,kw OR ward*:ti,ab,kw)) OR ED:ti,ab,kw OR ER:ti,ab,kw OR ((emergency:ti,ab,kw OR trauma:ti,ab,kw) NEAR/2 (care:ti,ab,kw OR doctor*:ti,ab,kw OR medicine:ti,ab,kw OR nurs*:ti,ab,kw OR patient*:ti,ab,kw OR physician*:ti,ab,kw OR personnel:ti,ab,kw OR provider*:ti,ab,kw OR psychiatr*:ti,ab,kw OR specialist:ti,ab,kw OR treatment*:ti,ab,kw)) OR emergentologist:ti,ab,kw OR emergicenter:ti,ab,kw OR "psychiatric EMS":ti,ab,kw)) AND ([mh "Pain Measurement"] OR [mh "Pain Management"] OR ("grimace scale":ti,ab,kw OR ((pain:ti,ab,kw OR analgesia:ti,ab,kw) NEAR/1 (assess*:ti,ab,kw OR intensity*:ti,ab,kw OR inventor*:ti,ab,kw OR measure*:ti,ab,kw OR question*:ti,ab,kw OR scale*:ti,ab,kw OR scor*:ti,ab,kw OR severity:ti,ab,kw OR test*:ti,ab,kw)) OR ("nociception" NEXT test*):ti,ab,kw OR "pain acceptance questionnaire":ti,ab,kw OR painDETECT:ti,ab,kw OR (VAS:ti,ab,kw NEAR/3 pain:ti,ab,kw) OR (("visual" NEXT analog* NEXT "scale"):ti,ab,kw AND pain:ti,ab,kw) OR "pain management":ti,ab,kw))

**CINAHL**

S1 (MH "Dementia+")

S2 (TI (alzheimer* OR 'diffuse cortical sclerosis' OR amentia* OR cadasil OR cadasilm OR ('cerebral arteriopathy' NEAR/2 'subcortical infarcts' ) OR 'cerebral autosomal dominant arteriopathy' OR (('basal cortical' OR basalcortical OR 'baso cortical' OR basocortical OR 'cortical basal' OR corticalbasal OR 'cortico basal' OR corticobasal ) N2 (degeneration OR syndrome )) OR CBD OR CBGD OR 'creutzfeldt jakob' OR CJD OR ((cognit* OR neurocognit* OR frontotemporal ) N2 (disorder* OR defect* OR deficit* OR decline* OR deteriorat* OR disabilit* OR dysfunction* OR disfunction* OR impair* OR interference* )) OR 'spongiform encephalopath*' OR dementia* OR demention OR ((aids OR hiv ) NEAR/3 (encephalopath* OR 'motor complex' )) OR huntington* OR 'kluver bucy syndrome' OR 'temporal lobectomy behavior syndrome' OR 'lewy bod*' OR lewybod* OR 'pick* complex' OR 'pick* disease' OR 'pick* syndrome' OR presenile OR 'prion* disease*' OR pseudodementia OR 'rett* disease' OR 'rett* syndrome' OR 'morbus rett' OR senile OR senilitas OR senility OR tauopathy*)) OR (AB (alzheimer* OR 'diffuse cortical sclerosis' OR amentia* OR cadasil OR cadasilm OR ('cerebral arteriopathy' NEAR/2 'subcortical infarcts' ) OR 'cerebral autosomal dominant arteriopathy' OR (('basal cortical' OR basalcortical OR 'baso cortical' OR basocortical OR 'cortical basal' OR corticalbasal OR 'cortico basal' OR corticobasal ) N2 (degeneration OR syndrome )) OR CBD OR CBGD OR 'creutzfeldt jakob' OR CJD OR ((cognit* OR neurocognit* OR frontotemporal ) N2 (disorder* OR defect* OR deficit* OR decline* OR deteriorat* OR disabilit* OR dysfunction* OR disfunction* OR impair* OR interference* )) OR 'spongiform encephalopath*' OR dementia* OR demention OR ((aids OR hiv ) NEAR/3 (encephalopath* OR 'motor complex' )) OR huntington* OR 'kluver bucy syndrome' OR 'temporal lobectomy behavior syndrome' OR 'lewy bod*' OR lewybod* OR 'pick* complex' OR 'pick* disease' OR 'pick* syndrome' OR presenile OR 'prion* disease*' OR pseudodementia OR 'rett* disease' OR 'rett* syndrome' OR 'morbus rett' OR senile OR senilitas OR senility OR tauopathy*))

S3 S1 OR S2

S4 (MH "Emergency Care") or (MH "Emergency Medical Services") or (MH "Emergency Services, Psychiatric") or (MH "Emergency Service+") or (MH "Emergency Nurses") or (MH "Emergency Nursing") or (MH "Emergency Treatment") or (MH "Emergency Medicine") or (MH "Emergency Nurse Practitioners") or (MH "Emergency Patients") or (MH "Physicians, Emergency")

S5 (TI ('acute care' OR 'acute medical care' OR (('a and e' OR a-e OR ae OR casualty OR emergency OR trauma ) N1 (center* OR centre* OR department* OR room* OR service* OR system* OR unit* OR ward* )) OR "ED" OR "ER" OR ((emergency OR trauma ) N2 (care OR doctor* OR medicine OR nurs* OR patient* OR physician* OR personnel OR provider* OR psychiatr* OR specialist OR treatment* )) OR emergentologist OR emergicenter OR 'psychiatric EMS') ) OR (AB ('acute care' OR 'acute medical care' OR (('a and e' OR a-e OR ae OR casualty OR emergency OR trauma ) N1 (center* OR centre* OR department* OR room* OR service* OR system* OR unit* OR ward* )) OR "ED" OR "ER" OR ((emergency OR trauma ) N2 (care OR doctor* OR medicine OR nurs* OR patient* OR physician* OR personnel OR provider* OR psychiatr* OR specialist OR treatment* )) OR emergentologist OR emergicenter OR 'psychiatric EMS') )

S6 S4 OR S5

S7 (MH "Pain Measurement") OR (MH "Pain Management")

S8 (TI ('grimace scale' OR ((pain OR analgesia ) N1 (assess* OR intensity* OR inventor* OR measure* OR question* OR scale* OR scor* OR severity OR test* )) OR 'nociception test*' OR 'pain acceptance questionnaire' OR painDETECT OR (VAS N3 pain ) OR ('visual analog* scale' AND pain ) OR 'pain management') ) OR (AB ('grimace scale' OR ((pain OR analgesia ) N1 (assess* OR intensity* OR inventor* OR measure* OR question* OR scale* OR scor* OR severity OR test* )) OR 'nociception test*' OR 'pain acceptance questionnaire' OR painDETECT OR (VAS N3 pain ) OR ('visual analog* scale' AND pain ) OR 'pain management') )

S9 S7 OR S8

S10 S3 AND S6 AND S9

**PsycInfo**

S1 DE "AIDS Dementia Complex" or DE "Alzheimer's Disease" or DE "Dementia with Lewy Bodies" or DE "Dementia" or DE "Frontotemporal Lobar Degeneration" or DE "Picks Disease" or DE "Presenile Dementia" or DE "Pseudodementia" or DE "Senile Dementia" or DE "Vascular Dementia"

S2 (TI (alzheimer* OR 'diffuse cortical sclerosis' OR amentia* OR cadasil OR cadasilm OR ('cerebral arteriopathy' NEAR/2 'subcortical infarcts' ) OR 'cerebral autosomal dominant arteriopathy' OR (('basal cortical' OR basalcortical OR 'baso cortical' OR basocortical OR 'cortical basal' OR corticalbasal OR 'cortico basal' OR corticobasal ) N2 (degeneration OR syndrome )) OR CBD OR CBGD OR 'creutzfeldt jakob' OR CJD OR ((cognit* OR neurocognit* OR frontotemporal ) N2 (disorder* OR defect* OR deficit* OR decline* OR deteriorat* OR disabilit* OR dysfunction* OR disfunction* OR impair* OR interference* )) OR 'spongiform encephalopath*' OR dementia* OR demention OR ((aids OR hiv ) NEAR/3 (encephalopath* OR 'motor complex' )) OR huntington* OR 'kluver bucy syndrome' OR 'temporal lobectomy behavior syndrome' OR 'lewy bod*' OR lewybod* OR 'pick* complex' OR 'pick* disease' OR 'pick* syndrome' OR presenile OR 'prion* disease*' OR pseudodementia OR 'rett* disease' OR 'rett* syndrome' OR 'morbus rett' OR senile OR senilitas OR senility OR tauopathy*)) OR (AB (alzheimer* OR 'diffuse cortical sclerosis' OR amentia* OR cadasil OR cadasilm OR ('cerebral arteriopathy' NEAR/2 'subcortical infarcts' ) OR 'cerebral autosomal dominant arteriopathy' OR (('basal cortical' OR basalcortical OR 'baso cortical' OR basocortical OR 'cortical basal' OR corticalbasal OR 'cortico basal' OR corticobasal ) N2 (degeneration OR syndrome )) OR CBD OR CBGD OR 'creutzfeldt jakob' OR CJD OR ((cognit* OR neurocognit* OR frontotemporal ) N2 (disorder* OR defect* OR deficit* OR decline* OR deteriorat* OR disabilit* OR dysfunction* OR disfunction* OR impair* OR interference* )) OR 'spongiform encephalopath*' OR dementia* OR demention OR ((aids OR hiv ) NEAR/3 (encephalopath* OR 'motor complex' )) OR huntington* OR 'kluver bucy syndrome' OR 'temporal lobectomy behavior syndrome' OR 'lewy bod*' OR lewybod* OR 'pick* complex' OR 'pick* disease' OR 'pick* syndrome' OR presenile OR 'prion* disease*' OR pseudodementia OR 'rett* disease' OR 'rett* syndrome' OR 'morbus rett' OR senile OR senilitas OR senility OR tauopathy*))

S3 S1 OR S2

S4 DE "Emergency Medicine" or DE "Emergency Medicine Physicians" or DE "Emergency Personnel"

S5 (TI ('acute care' OR 'acute medical care' OR (('a and e' OR a-e OR ae OR casualty OR emergency OR trauma ) N1 (center* OR centre* OR department* OR room* OR service* OR system* OR unit* OR ward* )) OR "ED" OR "ER" OR ((emergency OR trauma ) N2 (care OR doctor* OR medicine OR nurs* OR patient* OR physician* OR personnel OR provider* OR psychiatr* OR specialist OR treatment* )) OR emergentologist OR emergicenter OR 'psychiatric EMS') ) OR (AB ('acute care' OR 'acute medical care' OR (('a and e' OR a-e OR ae OR casualty OR emergency OR trauma ) N1 (center* OR centre* OR department* OR room* OR service* OR system* OR unit* OR ward* )) OR "ED" OR "ER" OR ((emergency OR trauma ) N2 (care OR doctor* OR medicine OR nurs* OR patient* OR physician* OR personnel OR provider* OR psychiatr* OR specialist OR treatment* )) OR emergentologist OR emergicenter OR 'psychiatric EMS') )

S6 S4 OR S5

S7 DE "Pain Management"

S8 (TI ('grimace scale' OR ((pain OR analgesia ) N1 (assess* OR intensity* OR inventor* OR measure* OR question* OR scale* OR scor* OR severity OR test* )) OR 'nociception test*' OR 'pain acceptance questionnaire' OR painDETECT OR (VAS N3 pain ) OR ('visual analog* scale' AND pain ) OR 'pain management') ) OR (AB ('grimace scale' OR ((pain OR analgesia ) N1 (assess* OR intensity* OR inventor* OR measure* OR question* OR scale* OR scor* OR severity OR test* )) OR 'nociception test*' OR 'pain acceptance questionnaire' OR painDETECT OR (VAS N3 pain ) OR ('visual analog* scale' AND pain ) OR 'pain management') )

S9 S7 OR S8

S10 S3 AND S6 AND S9

**ClinicalTrials.gov**

| Search iterations using Expert Search interface | |
| --- | --- |
| 1 | dementia AND (emergency OR ED OR ER) AND (“pain assessment” OR “pain intensity” OR “pain inventory” OR “pain inventories” OR “pain management” OR “pain measure” OR “pain measurement” OR “pain questionnaire” OR “pain scale” OR “pain scales” OR “pain severity” OR “pain test” OR “pain tests” OR “nociception test” OR “nociception tests” OR “grimace scale”) |
| 2 | alzheimer AND (emergency OR ED OR ER) AND (“pain assessment” OR “pain intensity” OR “pain inventory” OR “pain inventories” OR “pain management” OR “pain measure” OR “pain measurement” OR “pain questionnaire” OR “pain scale” OR “pain scales” OR “pain severity” OR “pain test” OR “pain tests” OR “nociception test” OR “nociception tests” OR “grimace scale”) |
| 3 | alzheimers AND (emergency OR ED OR ER) AND (“pain assessment” OR “pain intensity” OR “pain inventory” OR “pain inventories” OR “pain management” OR “pain measure” OR “pain measurement” OR “pain questionnaire” OR “pain scale” OR “pain scales” OR “pain severity” OR “pain test” OR “pain tests” OR “nociception test” OR “nociception tests” OR “grimace scale”) |
| 4 | alzheimer's AND (emergency OR ED OR ER) AND (“pain assessment” OR “pain intensity” OR “pain inventory” OR “pain inventories” OR “pain management” OR “pain measure” OR “pain measurement” OR “pain questionnaire” OR “pain scale” OR “pain scales” OR “pain severity” OR “pain test” OR “pain tests” OR “nociception test” OR “nociception tests” OR “grimace scale”) |

**World Health Organization International Clinical Trials Registry Platform**

| Search iterations using the basic search interface | |
| --- | --- |
| 1 | dementia AND emergency AND pain |
| 2 | dementia AND ED AND pain |
| 3 | alzheimer AND emergency AND pain |
| 4 | alzheimers AND emergency AND pain |
| 5 | alzheimer’s AND emergency AND pain |
